# Maternal and Neonatal Outcome following Day Two versus Day Five or Seven Discharge after an Uncomplicated Elective Caesarean Section: A Randomized Control Study

**DOI:** 10.1155/2021/9008772

**Published:** 2021-12-22

**Authors:** Fidelis A. Onu, Chidebe C. Anikwe, Johnbosco E. Mamah, Okechukwu B. Anozie, Osita S. Umeononihu, Bartholomew C. Okorochukwu, Ayodele A. Olaleye, John O. Egede, Cyril C. Ikeoha, Chigozie F. Okoroafor

**Affiliations:** ^1^Department of Obstetrics & Gynaecology, Alex Ekwueme Federal University Teaching Hospital Abakaliki, P.M.B. 102 Abakaliki, Ebonyi State, Nigeria; ^2^Department of Obstetrics and Gynecology, Nnamdi Azikiwe University Teaching Hospital, P.M.B. 5025 Nnewi, Anambra State, Nigeria; ^3^Department of Obstetrics and Gynecology, Federal Medical Centre Owerri, P.O. Box 1010 Owerri, Imo State, Nigeria

## Abstract

**Background:**

In recent times, it has become a common practice to discharge a woman early after an uncomplicated caesarean section (CS), to satisfy their wishes, reduce cost, and maximize efficient use of healthcare system resources.

**Objective:**

To conduct a comparative analysis of maternal and neonatal outcomes following day two hospital discharge versus day 5 or 7 discharge after an uncomplicated CS.

**Materials and Methods:**

Eligible parturient (228) who met the inclusion criteria were randomized into two groups between 1^st^ October 2018 and 30^th^ September 2019 in two different maternity centers in Ebonyi state. The study group (114) was discharged two days after an uncomplicated CS while the control group (114) was discharged on the 5^th^ or 7^th^ postoperative day. Their satisfaction, cost, morbidities, and breastfeeding practices were evaluated using a pretested questionnaire. Data were analyzed using IBM SPSS version 22.

**Results:**

Day 2 discharge was not associated with a higher rate of readmission as compared with day 5-7 discharge (*χ*^2^ = 0.95, *P* = 0.329). There were no statistically significant differences in cost incurred by patients discharged on day 2 after uncomplicated CS compared to the control group (*χ*^2^ = 1.65, *P* = 0.649). Maternal satisfaction was high following day 2 discharge compared with day 5-7 discharge (*χ*^2^ = 16.64, *P* = 0.0001, OR = 0.857, 95%CI = 0.59–1.25). The majority of mothers (79.6%) discharged on day 2 were able to initiate and sustain breastfeeding with no statistically significant difference in the initiation and sustenance of breastfeeding with those discharged on days 5-7 (*χ*^2^ = 4.45, *P* = 0.108). Early hospital discharge did not have any significant negative impact on neonatal health (*χ*^2^ = 1.063, *P* = 0.303).

**Conclusion:**

Early discharge of patients after an uncomplicated CS is not associated with increased rate of readmission. It is associated with good maternal satisfaction, adequate initiation and sustenance of breastfeeding, and good neonatal wellbeing. We advocate early discharge of women following uncomplicated CS.

## 1. Introduction

Patient satisfaction is a vital indicator of the quality of healthcare services available in a hospital [1]. In recent times, healthcare providers that endeavor to achieve excellence in patient care take into consideration patients' choices in devising strategies to achieve this objective [2]. One such strategy is reduced hospital stay [1, 2]. In obstetric practice, one of the common reasons for an extended hospital stay is caesarean section [3]. The length of stay depends on the indication for the procedure and individual patient characteristics [3]. This may range from 3 to 7 days in uncomplicated cases [3]. Studies done in our environment have shown that despite the safety of this procedure, patients have continued to exhibit a morbid aversion for caesarean section [4, 5]. The reasons for this aversion may be genuine, but in many cases, the fears are rather mythical [5]. A common reason for the aversion to caesarean section is due to rampant poverty [4–6]. Unpublished observations show that in Nigeria, caesarean deliveries cost between 100,000 and 500,000 naira depending on the facility and indication for the procedure [7]. This sum in most cases does not include medication fees and hospital admission fees. There is no gainsaying that over 70% of Nigerians live below the poverty line and Nigeria is ranked as 120^th^ among the lower-middle-income countries in the world [6]. In developing countries, out-of-pocket spending for healthcare services, worsened by the near absence of health insurance for the populace, has made this essential service nearly unaffordable to the people [7, 8].

There are other benefits to reduced hospital stay apart from cost reduction. Reduced hospital stay is negatively correlated with patient satisfaction [9, 10]. Indeed, patients who had shorter admission are more likely to be satisfied with the care received than patients who had extended hospital stay [10]. Studies equally showed that those who had reduced hospital stay were not more likely to be readmitted for any puerperal complications but they are more likely to continue breastfeeding at three-month follow-up [11].

Lack of social support is a recognized risk factor for puerperal psychiatric conditions such as maternal blues, depression, and psychosis. Early reintegration of a patient with her friends and family will ultimately attract love and support from all and sundry thereby reducing the physical and psychological stress on extended hospital stay which may be characterized by restricted activities including limited visitation periods from relatives [12].

Reduced hospital admission after an uncomplicated caesarean section also offers the advantage of allowing the parturient the freedom of ambulation; this will indirectly reduce the incidence of thromboembolic phenomenon associated with vascular stasis and coagulopathy due to prolonged immobility which may follow extended hospital admission [13].

The hospital-acquired infection continues to present a significant threat to patients on hospital admission. Such infections run a chronic course due to the high rate of antibiotic resistance associated with its treatment. Extended hospital admission will heighten the patients' risk of acquiring such infection; the risk is equally worse on her newborn who is particularly predisposed due to their immature immune system [13, 14]. Nosocomial (hospital-acquired infections) remains a major cause of perinatal morbidity and mortality.

Early hospital discharge will lead to increased infant-mother bonding due to rooming-in practice which is more likely in a calm serene environment away from the busy and sometimes noisy hospital wards prevalent in our setting. An enhanced infant-mother bonding offers significant advantages such as greater potential to sustain breastfeeding which improves the child's immunity and ultimate survival in an environment with high perinatal and neonatal mortality rates like ours [14, 15].

We believe that findings from this study would stimulate interest in this relatively unexplored aspect of patient care and lead to a change in current practice which is not evidence-based and does not improve overall patient outcome and satisfaction. More so, our finding will add to knowledge as no such study has been conducted on this subject in the study center.

## 2. Materials and Methods

### 2.1. Study Setting

This is a multicenter study conducted at the Alex Ekwueme Federal University Teaching Hospital Abakaliki (AE-FUTHA) and Mater Misericordiae Mission Hospital, Afikpo, between 1^st^ October 2018 and 30^th^ September 2019. These centers have specialist obstetrics and gynaecology departments with consultants, resident doctors, and midwives. They receive referral from general hospitals, mission hospitals, and primary health centers as well as privately owned hospitals and clinics. It also receives referral from neighbouring states.

### 2.2. Study Area

Ebonyi state is one of the five states in the southeast geopolitical zone of Nigeria. It has an estimated population of 2,176,947 people and occupies a landmass of 6400 km^2^, sharing boundaries in the west with Enugu state, Cross River in the south, and Benue state in the north [16]. Igbo is the predominant ethnic group in Ebonyi state, and the majority practice Christianity [16].

### 2.3. Study Population

Women included in the study were consenting women with uncomplicated elective antepartum caesarean section in our facility at term of a singleton pregnancy and residence in the study area. Uncomplicated elective antepartum caesarean section was defined as an elective delivery with no significant intraoperative complications such as primary postpartum haemorrhage requiring blood transfusion and other intervention, visceral injuries, and surgical site infection. The patient must be ambulating and had commenced oral feeds. Women excluded were women with two or more caesarean sections or with pregnancy complications such as uncontrolled hypertensive diseases of pregnancy, diabetes mellitus, sickle cell anaemia, and chorioamnionitis, no booking in our facility, the occurrence of perinatal death, and malformed fetuses.

### 2.4. Sample Size Calculation

Patient satisfaction will be used as the main outcome measure to be considered. Using proportions from the South African study by Buchmann et al. [17] based on the unmatched prospective cohort using patient satisfaction for early hospital discharge at 89.8% (*P*_0_ = 89.8%) and the unsatisfied patient rate for early hospital discharge at 10.2% (*P*_1_ = 10.2%),

(1) $$\begin{array}{c} n={\left(Z_{1-\alpha }+Z_{1-\beta }\right)}^{2}\frac{\left[P_{1}\left(1-P_{1}\right)P_{2}\left(1-P_{2}\right)\right]}{{\left(P_{1}-P_{2}\right)}^{2}}, \end{array}$$

where *n* is the required sample size, *Z*_1−*α*_ is the significance *α* level set at 5% (95%confidence interval = 1.96), *Z*_1−*β*_ is the power of the study *β* level set at 80% (2.56), *P*_1_ is the prevalence of the cases set at 89.8%, and *P*_2_ is the prevalence of the controls (expected to be 10% lower in satisfaction due to late discharge) set at 78.9%.

Substituting the values in the equation above, we got *n*_1_ = *n*_2_ = 94.8656 = 95 per arm.

Taking an attrition rate of 20%, we got 114 patients per arm—114 patients for day 2 group and 114 for day 5/7 group.

### 2.5. Patients' Education and Recruitment

Patients' education and selection were done at 36 weeks of gestation (antenatal clinic) when a birth plan of elective antepartum caesarean section was taken by her managing consultant. Patients selected were matched for age, parity, gestational age, and weight. The interval, from 36 weeks till delivery via elective caesarean section, afforded the women an opportunity to think the study through and discuss with their spouses. The study procedure was explained to the participants verbally in English or vernacular. The content of the health education included the purpose of the study, possible risks, and benefits. She was informed that if she consents to participate in the study, she will be randomly assigned to one arm of the study—either to day 2 discharge or to day 5 or 7 discharge and then followed up subsequently. They were informed that their phone numbers and contact addresses will be required for ease of communication and accessibility in the event of an emergency. Following the verbal explanation and demonstration of full understanding of the study, the patient was provided with a written consent form to go through, ask questions, and sign the consent form. Consecutive consenting patients were recruited using randomly generated numbers until the sample size was met. Patients selected were enrolled in the study and background information such as biodata and obstetrics characteristics—booking status, parity, number of deliveries, and outcome of previous childbirth/mode of delivery—were entered in an individualized coded study pro forma and coded. They were followed up till delivery when the labour outcome of her index pregnancy was entered.

### 2.6. Procedure for Randomization

The participants were randomized into two groups using computer-generated random numbers generated by the software Research Randomizer® (http://www.rando/http://mizer.org/). This was done at the antenatal ward when they were admitted for workup for caesarean section. Using this software, one hundred and fourteen numbers (114) were randomly generated from a pool of two hundred and twenty-eight (228) numbers, and these numbers were assigned to the study group (day 2 discharge). The remaining 114 numbers were automatically assigned to the control group (day 5/7 discharge). These numbers were inscribed on a brown envelope, and a piece of paper with the inscription study group/day 2 group or control group/day 5/7 discharge was placed inside the respective envelopes and sealed. Participants that met the inclusion criteria who have signed the informed consent form were given a sequential study number, and the corresponding numbered opaque sealed envelopes were then allocated to the patient. Women however selected but whose delivery was complicated by significant intraoperative complications such as primary postpartum haemorrhage requiring blood transfusion and other intervention, visceral injuries, admission in intensive care unit (ICU), neonatal admission in ICU, and perinatal death were removed from the study. New parturient was selected.

### 2.7. Outcome Measures

The primary outcome includes patient satisfaction with the discharge protocol used, cost of hospital care during and after admission, and rate of readmission or unscheduled medical consultation for any complaint. Sustenance of breastfeeding at 6^th^ week postoperative and willingness to go through the same protocol in the next delivery were the secondary outcomes.

### 2.8. Study Intervention

All eligible women undergoing elective CS who have signed the informed consent form were randomized. A pro forma was filled by the researcher for each of the eligible women who consented to the study.

#### 2.8.1. Study Group

This consists of patients who were discharged on the second day following surgery. Discharge was based on their meeting the requirements on the eligibility checklist (Appendix I).

#### 2.8.2. Control Group

This group included patients that were discharged between days 5 and 7 depending on the surgical characteristics vis-à-vis subcuticular skin closure that was discharged on the fifth day while interrupted mattresses were discharged on day 7.

### 2.9. Procedure for the Intervention

The study population was divided into two groups: the study group and the control group. Each recruited participant in the study group was pair-matched with control who also had an elective uncomplicated caesarean section. Following day two postop discharge, recruited patients in the study group were followed up by the researcher or trained research assistants with twice-daily phone calls, in the mornings and evening. During each interaction with the parturient, the inquiries made included the patients' general state of health, any postoperative site pains, any fever, bleeding per vaginam, breastfeeding, and neonatal wellbeing and other concerns of the patient were explored and addressed. Any complaint that required a hospital assessment required the parturient to present to the hospital for expert review. In the absence of major complaints, this regular phone monitoring was continued until the fifth or seventh day depending on when the matching patient on the control arm was discharged. This is to ensure that the patient in the study group receives an equal length of medical attention with the matching pair on the control group without necessarily being on hospital admission. Patients were encouraged to call the researcher and research assistants at any time of the day when the need arose.

### 2.10. Departmental Protocol for Caesarean Section and Postoperative Management of Uncomplicated Elective Caesarean Section

Women scheduled for elective caesarean section are admitted a day prior to the D-day. Elective caesarean section is carried out at 38 weeks and above in our department. Women admitted for caesarean section will have a detailed history taking, physical examination, and investigations. The following investigation are done—biophysical profile, full blood count, urinalysis, random blood sugar, kidney function test, and blood grouping/cross matching of two (2) units of blood. The anesthetist team on call is invited to review her. The pediatrician is also invited to be present at the theater during the delivery for neonatal resuscitation. Following uncomplicated elective caesarean section, it is our practice to maintain a high dose oxytocin infusion for 12 to 24 hours against primary postpartum haemorrhage. Parenteral antibiotics are administered for 48 hours postcaesarean section and continued on oral antibiotics. Oral intake of fluids and feeds is commenced between 12 and 24 hours postsurgery, urethral catheters are removed between 24 hours after surgery, and patients are encouraged to ambulate as soon as they recover from anesthesia. Wound inspection and assessment of postoperative packed cell volume are done 48 hours postoperative. Mothers are encouraged to commence breastfeeding following full recovery from anesthesia. The women are observed for a further 3 to 5 days after which they are discharged between the 5^th^ and 7^th^ day postop. Women who had subcuticular skin closure are discharged on the 5^th^ day while those who had interrupted mattress skin closure would have sutures removed on the 7^th^ day after which they are discharged home. Except otherwise indicated, discharged women are seen two weeks postsurgery to assess their adaptation at home and for wound inspection. In the absence of anomaly, they are given a further appointment, coincident with their 6^th^ week postnatal visit.

### 2.11. Data Collection

Data were collected by the principal researcher or the research assistants using a pretested structured questionnaire, which was administered verbally to the study subjects in the postnatal ward. Information obtained included sociodemographic details, nature of surgery, indications, fetal outcome, commencement and tolerance of oral intake, and attainment of other relevant criteria as specified for eligibility. Completed questionnaires were checked for completeness, consistency, and accuracy. They were coded and entered into a password-protected Microsoft Excel Database.

### 2.12. Data Analysis

All data analysis was carried out using a statistical package for social sciences version 20 (IBM-SPSS version 20). Descriptive statistics were used for sociodemographic characteristics. Where applicable, results were expressed as mean and standard deviation (mean ± SD). The Pearson correlation coefficient was used to measure the degree of association between variables. Chi-square test (*χ*^2^) was used to compare means of categorical variables, and the Student *t*-test was used to compare means of a continuous variable. The significance level was at a *P* value of <0.05 at a 95% confidence interval.

Patient satisfaction and associated adverse outcomes were estimated using simple proportions. A four-point Likert scale (strongly agree, agree, disagree, and strongly disagree) was used to assess the maternal satisfaction to the day of discharge, recommend protocol to a friend, and maternal wellbeing. A study participant whose responses were “strongly agree or agree” is classified as having positive disposition to the outcome measure while women whose responses were “disagree or strongly disagree” are classified as having negative disposition to the measure. Maternal satisfaction to the day of discharge was classified into “satisfied” (yes—positive) and “not satisfied” (no—negative), recommendation of protocol into “yes—positive” and “no—negative,” and maternal wellbeing into “good—positive” and “fair—negative.” Maternal satisfaction was assessed using a satisfaction questionnaire adapted from previous study in the hospital [18]. It is composed of 20 satisfaction-related variables which came up with a high internal consistency (Cronbach's alpha = 0.82). The satisfaction variables were grouped into four categories: healthcare (four questions), health worker communication (six questions), attitude of the health workers (six questions), and the hospital physical environment (four questions). The maternal assessment of caesarean section experience was evaluated using the four-point Likert scale as explained above. To assess women's overall satisfaction with the quality of hospital care, the summary section of the questionnaire contained three indicators which included one direct and two indirect summary questions asked against the background of women's responses to previous inquiries on the various aspects of hospital care. It would be expected that this “overall satisfaction” variable would reflect women's overall perception of the quality of care received. This variable was determined by respondents' affirmative answers to these three questions: “Are you satisfied with the day of discharge?,” “Would you recommend protocol to somebody else?,” and “In general, are you satisfied with the care you have received in the hospital?” For the purpose of this study, an affirmative answer to all of the three questions by the respondent was considered an index of true maternal satisfaction with care received.

Cost was assessed by calculating the total cost of care for each participant—the study group versus the control group. Breastfeeding was initially assessed during the two weeks' visit to the hospital and later at six weeks' postnatal care. For those that were discharged earlier, daily calls enable us to ascertain any difficulty with breastfeeding. Affirmative response by a participant that the neonate is exclusively breast fed—assessed by adequate neonatal weight gain—is used as an evidence of adequate initiation and sustenance of breastfeeding.

### 2.13. Ethical Considerations

Permission to carry out this research was obtained from the Research and Ethics Committee of AE-FUTHA from 14^th^ November 2017 to 19^th^ December 2017 (approval number is AE-FETHA/REC/VOL/1/2017/631). The trial was registered with the Pan African Clinical Trial Registry with the trial number PACTR 202105761282607. Informed and written consent was obtained from the women before they were included in the study.

## 3. Results

**Figure 1:**
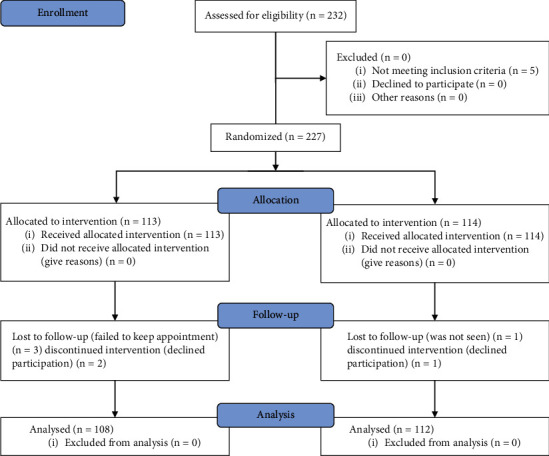
Flowchart of patients through the study.

As depicted in Figure 1, 232 cases of uncomplicated elective caesarean sections were managed during the study period. These patients were randomized into two groups, making 116 patients in each arm of the study. Of the 116 cases randomized, 113 met the inclusion criteria and were recruited. Three patients out of the 113 that met inclusion and recruited declined further participation and were excluded from the study, and another 2 patients were lost to follow-up. The remaining 108 cases were analyzed. In the control group, 114 met the inclusion criteria and were recruited. One patient in the control group declined further participation and was excluded from the study, and another patient was lost to follow-up. The remaining 112 patients in the control group were analyzed.

Table 1 represents the sociodemographic and baseline clinical characteristics of both groups of patients. There was no statistically significant difference in these parameters between both groups of study participants.

Table 2 compares the surgical profile of both groups of patients. There were no statistically significant differences in the surgical profile of both groups of participants.

Table 3 compares postoperative complications experienced by parturient discharged on day 2 postoperation with the complications experienced by parturient discharged on days 5-7 postoperation. Discharge of a patient on day 2 postoperation does not significantly increase the risk of complications. However, discharge of patients on day 2 postoperation significantly increased the risk of postoperative pains experienced by parturient.

In Table 4, neonatal morbidities and breastfeeding practices of parturient discharged on day 2 postoperation were compared with those discharged on days 5-7 postoperation. Discharge of a patient on day 2 postoperation did not significantly increase the risk of neonatal morbidities and breast morbidities. Early discharge of parturient on day 2 postoperation did not have any significant effect on breastfeeding practices as compared with parturient discharged on traditional days 5-7 postoperation.

Table 5 compares the perception of mothers on the days of their discharge after caesarean section. The majority of parturient discharged on day 2 postoperation were satisfied with the days of their discharge, have a positive perception of their wellbeing after discharge, and are ready to recommend the protocol to their friends, as compared to those discharged on days 5-7 postoperation.

## 4. Discussion

In obstetric practice, one of the most common reasons for extended hospital stay is caesarean section. The length of stay depends on the indication for the procedure and individual patient characteristics which may range from 3 to 7 days in uncomplicated cases [3]. However, reduced hospital stay has been shown to be cost-effective [7, 8], negatively correlated with patients' satisfaction [9, 10], reduced puerperal complication, early reintegration of parturient into community to reduce psychiatric complications, reduce the incidence of thromboembolic phenomenon, reduced risk of nosocomial infections, and improved infant-mother bonding.

From this study, it can be inferred that discharge of a patient on day 2 postoperation did not significantly increase the rate of readmission or unscheduled medical consultation for any complication for both the mother and her fetus as compared to traditional methods of discharging patients on day 5-7 postoperation. This finding is in keeping with what Thomas in Mulago Hospital in Uganda discovered as part of secondary outcome of his study that there was no statistically significantly difference in the rate of readmission among parturient discharge on day 2 as compared to those discharged on days 5-7 [19]. However, in a study done by Umbeli et al. [20], it was discovered that the rate of readmission was higher in the control group who were discharged on the traditional days 5-7, as opposed to those discharged on day 2 postoperation. This trend was attributed to increased rate of wound infection and deep vein thrombosis which was commoner among the control group. In a related study by Ghaffari et al. in which women were allowed to go home on the first compared with the second day after a planned caesarean delivery, the maternal postpartum outcomes were found to be similar one week and 6 weeks postpartum [21]. Day 1 discharge might be difficult to advocate in the study area because of the pervading poor maternal and child healthcare services in Nigeria. It, however, calls for astute patient selection and in a good clime before its implementation.

This study also demonstrated that there was no significant difference in the cost analysis of day 2 hospital discharge as compared to day 5-7 hospital discharge after an uncomplicated elective caesarean section. This may be due to the fact that majority of patients that had uncomplicated elective caesarean section were on National Health Insurance Scheme that subsidized their hospital bills. However, Brumfield et al. reported that day 2 discharge helped saved 440 dollars per patient as compared to traditional discharge after days 5-7 [22]. Thomas in Mulago Hospital reported that early discharge has significant cost-saving effects on the participants [19]. Also, Grullon and Grimes reported that early discharge with subsequent follow-up has significant cost-lowering effect when compared with traditional day 5-7 discharge [23]. Sainz Bueno et al. stated that early hospital discharge has 20% reduction in the cost of treatment [24].

Our study showed that the level of maternal satisfaction following day 2 hospital discharge as compared to the traditional day 5-7 discharge after an uncomplicated elective caesarean section is statistically significant (OR = 0.857, 95%CI = 0.59–1.250). Similar finding was reported by Tan et al. [25]. Umbeli et al. in Sudan reported that 85.6% of the women in the study group were satisfied with the early discharge, while only about 37.2% of the women in the control group were satisfied with the prolonged hospital stay [20]. Similarly, Norton in Reuters [26] and Todd [27] in Steady Health reported that more women are satisfied with early discharge after uncomplicated caesarean section as opposed to traditional day 5-7 discharge. In a cohort study conducted by Buchmann et al. in South Africa, he reported that early discharge is safe and more satisfying to the patient, with 89.8% of the patients suggesting they would choose it again [17].

In our study, there was no statistically significant difference in the rate of initiation and sustenance of breastfeeding among women discharged on the second day following an uncomplicated elective caesarean section as compared with those discharged on days five to seven. This may be due to the fact that the uncomplicated nature of indications for caesarean section encourages early recovery and subsequent commencement and sustenance of breastfeeding on both arms of study. This finding is similar to the outcome of the study done by Tan et al. [25] who reported that patients' satisfaction and commencement of exclusive breastfeeding are comparable between both cases and control. Also, in a randomized controlled trial in Switzerland, it was found that there was no significant difference between the groups of patients discharged on day 2 as opposed to those discharged on days 5-7 regarding the initiation and maintenance of breastfeeding practices [23]. However, in a study done in Sweden and in a randomized controlled trial done in Canada, the researchers found that those who had early discharge were more likely to practice and sustain exclusive breastfeeding compared with those that had routine hospital care [28, 29]. The differences in findings may be due to cultural differences in breastfeeding practices.

Also, early hospital discharge did not have any statistically significant negative impact on neonatal health and did not significantly increase the rate of neonatal readmission among the two groups of women we studied. A plausible reason of the above finding might not be unrelated to the fact that majority of women randomized on both arms of the study are young and fit, which subsequently lead to favourable outcome on their fetuses. In conclusion, early discharge of patients after elective caesarean section did not significantly increase the risk of readmission or unscheduled medical consultation for any complication; it is not associated with untoward maternal satisfaction and initiation and sustenance of breastfeeding and has no negative effects on neonatal health or risk of neonatal readmission. Although, from our findings, there was no significant cost-saving effect of this practice, this can be attributed to the widespread use of National Health Insurance Scheme among our respondents.

### 4.1. Strengths of the Study

Randomization and blinding were achieved satisfactorily. Data collection was done without bias by an independent person whose is not part of the authors. It is a multicenter study, and this helps to ensure better coverage and strengthened the validity of the result.

### 4.2. Limitation of the Study

Some follow-up information obtained through phone calls is subject to recall bias, and this may affect the validity of the information. It could also cause social desirability bias. We tried circumventing it through adequate counseling of study participants on the need to be frank with their responses and also by giving the women two weeks' appointment to the clinic. This assisted us to generally assess the maternal and neonatal wellbeing vis-à-vis their responses.

### 4.3. Conclusion and Recommendations

This study has demonstrated that early hospital discharge of patients is associated with untoward maternal satisfaction, adequate initiation, and sustenance of breastfeeding and did not significantly increase the risk of readmission or unscheduled medical/surgical consultations for the mother and her fetus. The early discharge did not significantly increase postoperative complications and could be cost-effective in certain settings. It is therefore recommended that day 2 discharge after an uncomplicated elective caesarean section be included as part of our departmental protocols for management of patients undergoing uncomplicated elective caesarean sections. Further studies on this subject matter are encouraged to fully outline all the advantages associated with early hospital discharge after an uncomplicated elective caesarean section.

## Figures and Tables

**Table 1:** Social and demographic features of the women.

Parameter	Day 2 discharge (*n* = 108) (%)	Day 5-7 discharge (*n* = 112) (%)	*χ* ^2^	*P* value
Age			1.89	0.596
<20	1 (0.9)	2 (1.8)		
20-29	45 (41.7)	54 (48.2)		
30-39	58 (53.7)	54 (48.2)		
≥40	4 (3.7)	2 (1.8)		
Parity			4.65	0.098
0	3 (2.7)	6 (5.4)		
1-4	103 (95.4)	98 (87.5)		
≥5	2 (1.9)	8 (7.1)		
Marital status			3.95	0.139
Married	104 (96.3)	106 (94.6)		
Single	2 (1.9)	6 (5.4)		
Widowed	2 (1.9)	0 (0.0)		
Occupation			5.47	0.242
None	26 (24.1)	26 (23.2)		
Farming	8 (1.9)	8 (7.1)		
C/S	48 (44.4)	36 (32.1)		
Trading	24 (22.2)	38 (33.9)		
Artisan	2 (7.4)	4 (3.6)		
Education			0.57	0.753
Primary	16 (14.8)	20 (17.9)		
Secondary	43 (39.8)	46 (41.1)		
Tertiary	49 (45.4)	46 (41.1)		
Tribe			2.42	0.120
Igbo	96 (88.9)	106 (94.6)		
Others	12 (11.1)	6 (5.4)		
Gestational age			3.19	0.364
≥36	14 (13.0)	10 (8.9)		
37-38	36 (33.3)	40 (35.7)		
39-40	52 (48.1)	58 (51.8)		
≥41	6 (5.6)	4 (3.6)		
Religion			0.001	0.971
Christian	106 (98.1)	110 (98.2)		
Others	2 (1.9)	2 (1.8)		

**Table 2:** Surgical profile of the study population.

Parameters	Day 2 discharge (*n* = 108) (%)	Day 5-7 discharge (*n* = 112) (%)	*χ* ^2^	*P* value
Surgical time			1.04	0.596
<1 hour	28 (25.9)	36 (32.1)		
1-2 hours	76 (70.4)	72 (64.3)		
>2 hours	4 (3.7)	4 (3.6)		
Cadre of surgeon^∗∗^			4.69	0.096
Consultant obstetrician	48 (44.4)	66 (58.9)		
Senior registrar	58 (53.7)	44 (39.3)		
Registrar	2 (1.9)	2 (1.8)		
Anesthesia			0.59	0.445
Regional	66 (61.1)	74 (66.1)		
General	42 (38.9)	38 (33.9)		
Skin incision			3.33	0.068
Midline	16 (14.8)	8 (7.1)		
Transverse	92 (85.2)	104 (92.9)		
Skin closure			1.26	0.262
Interrupted	4 (3.7)	8 (7.1)		
Subcuticular	104 (96.3)	104 (92.9)		
Blood loss			3.06	0.217
<500 ml	41 (38.0)	31 (27.7)		
500-1000 ml	63 (58.3)	78 (69.6)		
>1000 ml	4 (3.7)	3 (2.7)		
Cost of care (naira)^‡^			1.65	0.649
<50,000	1 (0.9)	1 (0.9)		
50-100,000	21 (19.4)	19 (17.0)		
101-150,000	76 (70.4)	86 (76.8)		
>150,000	10 (9.3)	6 (5.4)		

^∗^Consultant obstetrician—specialist; senior registrar—specialist in training who has passed his 1^st^ tier of examination but yet to pass his last tier of examination; registrar—a medical doctor in training who is yet to pass any tier of examination ^‡^360 naira = 1 USA dollar.

**Table 3:** Maternal complication among the women.

Variable	Day 2 discharge	Day 5-7 discharge	*χ* ^2^	*P* value
Complication			1.946	0.496
Fever	0	2		
Wound discharge	0	2		
Wound breakdown	0	1		
Bleeding from wound	0	1		
Pain at the wound	3	2		
Abdominal pain	0	0		
Offensive vagina discharge	3	6		
Psychiatric disorder	0	0		
Unwell	0	0		
Excessive vaginal bleeding	4	3		
Postdischarge consultation			0.412	0.521
Mother	20 (18.51)	18 (16.1)		
Neonate	4 (3.7)	2 (1.8)		
Readmission			0.95	0.329
Mother	6 (5.6)	8 (7.1)		
Neonate	4 (3.7)	2 (1.8)		
Grading of pain			19.63	0.0002
No pain	10 (9.3)	4 (3.6)		
Mild pain	86 (79.6)	78 (69.6)		
Moderate pain	8 (7.4)	30 (26.8)		
Severe pain	4 (3.7)	0 (0.0)		

**Table 4:** Neonatal morbidities and breastfeeding practices.

Parameters	Day 2 discharge (*n* = 108) (%)	Day 5-7 discharge (*n* = 112) (%)	*χ* ^2^	*P* value
Neonatal morbidities			1.063	0.3025
No problem	96	88		
Fever	6	12		
Yellow eyes	2	10		
Purulent vaginal discharge	0	0		
Vomiting	0	0		
Passage of watery stool	0	0		
Readmission	4	2		
Breastfeeding practice			4.45	0.108
Exclusive	86 (79.6)	96 (85.7)		
Mixed	18 (16.7)	16 (14.3)		
Breast milk substitute	4 (3.7)	0 (0.0)		
Breast morbidities			0.77	0.856
Cracked nipples	4 (3.7)	4 (3.6)		
Engorged breast	4 (3.7)	2 (1.8)		
Mastitis	2 (1.9)	2 (1.8)		
None	98 (90.7)	104 (92.9)		

**Table 5:** Maternal perception of the day of discharge.

Parameters	Day 2 discharge (*n* = 108) (%)	Day 5-7 discharge (*n* = 112) (%)	*χ* ^2^	*P* value	OR (95% CI)
Satisfied with the day of discharge					
Yes	108 (100)	96 (76.8)	16.64	0.0001	0.857 (0.59-1.250)
No	0 (0.0)	16 (23.2)	Ref.		
Recommend protocol to a friend					
Yes	104 (96.3)	50 (44.6)	34.93	0.0001	0.46 (0.30-0.71)
No	4 (3.7)	62 (56.4)	Ref.		
Maternal wellbeing					
Good	98 (90.7)	92 (82.1)	Ref.		
Fair	10 (9.3)	20 (17.9)	3.45	0.063	0.91 (0.61-1.33)

## Data Availability

All data generated or analyzed during this study are included in this published article.

## Appendix

1. Uncomplicated singleton elective caesarean section
2. Pain not more than mild at rest or moderate on movement
3. Ability to walk without difficulty
4. Lactating satisfactorily
5. Urinate without difficulty
6. Eating/drinking normally
7. Satisfactory wound status
8. Normal vital signs
9. Normal lochia
10. Satisfactory mental state assessment
11. Postoperative pack cell volume ≥ 10 g/dl
12. Have a reliable good support at home
13. Have a reliable means and access to the hospital
14. Have a valid address and functional phone number
15. Have adequate postoperative medications to last till next appointment
16. Have been counseled on infant feeding (exclusive breastfeeding)
17. Neonate suckling (breastfeeding) satisfactorily
18. No concern about mother and newborn health
